# A Case of Myomatous Erythrocytosis Syndrome Associated with a Large Uterine Leiomyoma

**DOI:** 10.1155/2014/602139

**Published:** 2014-01-14

**Authors:** Yosuke Ono, Takao Hidaka, Kaori Fukuta, Keiko Kouchi, Kuniaki Yasoshima, Kiyoshi Takagawa, Takashi Arai

**Affiliations:** ^1^Department of Obstetrics and Gynecology, Kurobe City Hospital, Mikkaichi 1108-1, Kurobe, Toyama, Japan; ^2^Department of Obstetrics and Gynecology, University of Toyama, Sugitani 2630, Toyama, Toyama, Japan; ^3^Department of Pathology, Kurobe City Hospital, Mikkaichi 1108-1, Kurobe, Toyama, Japan

## Abstract

Several etiologies have been proposed for erythrocytosis associated with uterine leiomyoma. We report a case of erythrocytosis associated with a large uterine leiomyoma, in which specific immunostaining for erythropoietin was positive. A 55-year-old woman, gravida 0, para 0, was referred to our hospital for treatment for a large uterine myoma and erythrocytosis. She had no vaginal bleeding after she reached menopause at 50 years old. She had severe polycythemia: hemoglobin (Hb), 19.9 g/dL; red blood cell count (RBC), 6.65 × 10^6^/mm^3^; hematocrit, (Hct) 59.1%. An abdominal simple hysterectomy was performed, and a pathological examination confirmed the diagnosis of leiomyoma of the uterus. In addition, immunostaining demonstrated that the cytoplasm of the leiomyoma cells was strongly positive for erythropoietin. After the operation, the patient's hemoglobin and hematocrit levels normalized, and we diagnosed her condition as myomatous erythrocytosis syndrome.

## 1. Introduction

Ectopic erythropoietin production is known to occur as a complication of various tumors such as renal cell carcinoma, hepatocellular carcinoma, and cerebellar hemangioblastoma. However, it is less well known that it can also occur as a complication of uterine leiomyoma.

Since Thomson and Marson first described myomatous erythrocytosis syndrome in 1953 [1], the etiology of the syndrome has been investigated using a range of methods, and various etiologies have been proposed [2, 3].

## 2. Case

**Figure 1:**
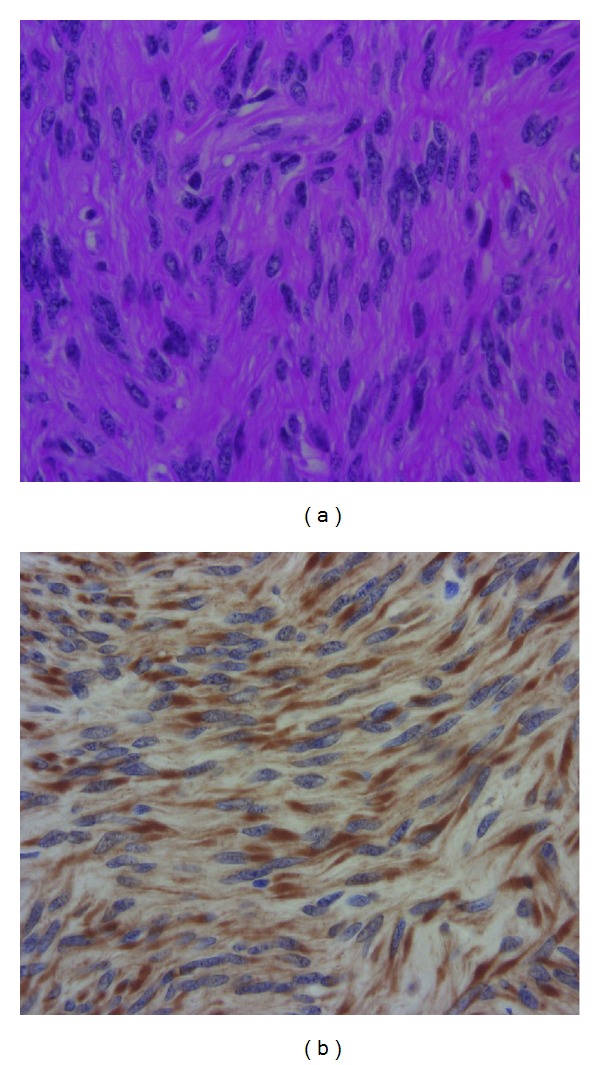
(a) Hematoxylin-eosin stain (HE stain). Uniform, spindle-shaped, smooth muscle cells are shown, which were considered to be uterine leiomyoma cells. (b) Specific immunostaining for erythropoietin was detected in the cytoplasm of the leiomyoma cells. The cytoplasm of most leiomyoma cells was strongly stained.

A 55-year-old woman, gravida 0, para 0, was referred to our department for treatment for a large uterine myoma. She presented with hyperhemoglobinemia, and hematological examinations produced the following results: hemoglobin, 19.9 g/dL; hematocrit, 59.1%; white blood cell count, 7,900/mm^3^; red blood cell count, 6.65 × 10^6^/mm^3^; and serum levels of erythropoietin, 28.5 mU/mL (normal range: 0–29.0 mU/mL). In pelvic examination, uterus size was newborn child head, the mobility of uterus was good, and she had no abdominal tenderness. The size of uterus was over 20 cm. And there was a giant intramural myoma which was cm × cm, in preoperative pelvic ultrasound examination. The result of preoperative cytology of the endometrium was normal though the preoperative diagnostic endometrial curettage was not performed. Her cardiovascular and respiratory systems were normal. The results of other examinations, including magnetic resonance imaging (MRI) of the head to find the cerebellar hemangioma which could produces erythropoietin, upper abdominal ultrasonography, and chest X-rays were all normal. We suspected myomatous erythrocytosis syndrome and hysterectomy was planned. To normalize the patient's condition, 1,600 mL of blood were removed by phlebotomy 4 times before surgery. As a result, her hemoglobin level decreased to 14.9 g/dL, and her hematocrit level fell to 49.8%. Abdominal total hysterectomy was performed, which resulted in approximately 510 mL of blood loss. The weight of the uterus, including the myoma node, was 1,280 g, and a histological examination confirmed the diagnosis of leiomyoma (Figure 1). In order to confirm that the uterine leiomyoma was producing erythropoietin, the expression of erythropoietin in the tumor was investigated immunohistochemically. Immunostaining for erythropoietin was performed using the indirect method and the EPO (N-19):SC-1310 antibody (Santa cruz biotechnology, inc.). As a result, it was found that the cytoplasm of the leiomyoma cells was strongly positive for erythropoietin.

The patient was discharged from hospital on the sixth postoperative day with no symptoms. At 4 postoperative weeks, her hemoglobin level had normalized to 12.7 g/dL, her hematocrit level had fallen to 37.6%, and her serum erythropoietin concentration had decreased from 28.5 mU/mL to 25.9 mU/mL. At 11 postoperative months, she is currently being followed-up, and her blood cell counts remain normal (hemoglobin, 12.7 g/dL; hematocrit, 37.6%).

## 3. Discussion

The first case of myomatous erythrocytosis syndrome was reported in 1953 by Thomson and Marson [1]. In terms of its effects on the blood, myomatous erythrocytosis syndrome only involves an elevated red blood cell count and does not exhibit the pancytosis that characterizes polycythemia [2, 3]. In 1957, three diagnostic criteria for myomatous erythrocytosis syndrome were proposed: (1) erythrocytosis, (2) a myomatous uterus, and (3) the restoration and maintenance of normal hematological values after hysterectomy [4]. Our case fulfilled these criteria. Various mechanisms have been proposed to cause the erythrocytosis encountered in myomatous erythrocytosis syndrome, for example, both intrauterine shunting and myoma have been suggested as causative factors [3–7]. However, the actual mechanism remains unclear. The hypothesis that uterine myomas produce erythropoietin in an autonomous manner and are not subjected to negative feedback control mechanisms is currently the most credible and plausible explanation. Recently, Suzuki et al. reported a case of myomatous erythrocytosis syndrome, in which they evaluated the level of erythropoietin in leiomyoma tissue using a radioimmunoassay [8]. In addition, Kohama et al. reported a case in which erythropoietin messenger RNA in leiomyoma tissue was detected by the reverse-transcription polymerase chain reaction (RT-PCR) and southern blot analysis [6]. These studies were the first to assess myomatous erythrocytosis syndrome using molecular biological techniques. On the other hand, Yokoyama et al. first detected erythropoietin in the leiomyoma tissue of patients with myomatous erythrocytosis syndrome using an immunohistochemical method [7]. In our case, the serum level of erythropoietin before surgery was within the upper limit of normal. Suzuki et al. reported that the serum levels of erythropoietin in their case were within the normal limits [8]. Therefore, although leiomyomas produce erythropoietin, the serum erythropoietin level might not always be a reliable indicator of the degree of erythrocytosis.

We experienced a case of myomatous erythrocytosis syndrome, which was diagnosed by detecting erythropoietin in a leiomyoma using an immunohistochemical method. In daily practice, clinicians should be aware that hysterectomy might improve polycythemia in patients with uterine leiomyoma.
